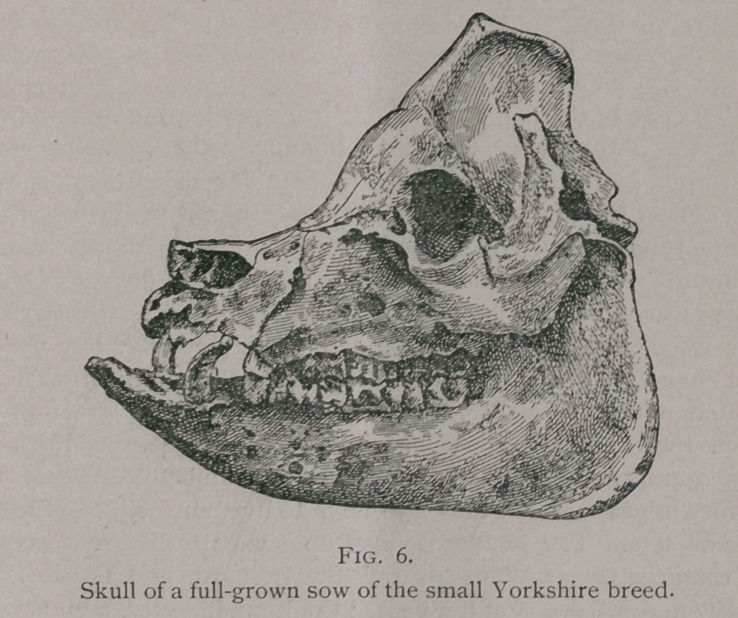# Age of the Hog

**Published:** 1891-12

**Authors:** R. S. Huidekoper

**Affiliations:** Vet.


### AGE OF THE HOG.*

The age of the hog is of much less importance than that of any of the domestic animals, as this animal becomes an article of commerce and is sent to the butcher the moment that the ratio of its increase of weight (flesh and fat) ceases to be greater than the proportionate cost of the food which is furnished to it. Even for breeding purposes, the hog is rarely kept to an advanced age. The question of its age only becomes important in exhibitions, where the judges must verify the age in the class for which the animal is entered.

---

From "Age of the Domestic Animals," by R. S. Huidekoper, M. D., Vet. Just published.

DENTITION.

Formula { Temporary, . . . $\frac{3 \cdot 1 \cdot 3}{3 \cdot 1 \cdot 3} = 28$

Permanent, . . . $\frac{3 \cdot 1 \cdot 4 \cdot 3}{3 \cdot 1 \cdot 4 \cdot 3} = 44$

FIG. 1.

Low jaw of pig, showing permanent dentition.

The hog has forty-four teeth, six incisors in each jaw (12), two tushes in each jaw (4) and seven molars (which are subdivided into four premolars and three molars) in each arch of both jaws (28).

INCISORS.

The pinchers and intermediate teeth of the upper jaw are flattened from side to side, somewhat like those of the horse, as they also have a small dental cup; while those of the lower jaw are straight, rounded, and directed forward like the teeth of a rodent, with their extremities converging toward the median line. The corner teeth of both jaws are small, conical teeth, separated from the intermediate on the one side and from the tusks on the other. They have no cups, but often have three small tubercles like the incisors of the dog.

### Tusks.

The tusks are much more developed in the male than in the female; they are prismatic in shape and curve outward and backward. In the wild hog they may attain an enormous length (six inches or more), pressing the upper lip upward and the lower lip downward and crossing each other in an X, the superior tusk passing behind the inferior; the two frequently have their respective anterior and posterior faces much worn by the constant friction from their contact with each other. The tusks continue to grow throughout the life of the animal. The growth is diminished in the castrated male.

### Molars.

The molars are divided into premolars and post-molars. The first premolar resembles very much the conical corner incisor tooth, but has two roots instead of one. It is placed in the interspace between the tusks and the other molars, being about twice as far from the latter as from the former.

The other molars are between the inferior molars of the horse and the molars of the dog in form. They wear by the centre of the crown and have not the irregular asperities found in the herbivora. They increase gradually in size from in front to behind, and are larger in the upper jaw than in the lower.

### Determination of Age by the Teeth.

To Dr. Olof. Schwartzkopff I am indebted for the following:

"During the past few years many objections have been raised, on the part of our practical breeders, to the correctness of the older rules for recognizing the age of our domestic animals. Several cases of an extraordinarily early development of the dentition have been observed in fat-stock shows and other exhibitions, and it has been alleged that modern feeding, with the tendency to produce early maturity, results also in an earlier shedding of the teeth. Not only in the United States have these doubts been heard, but also in England and Germany. In 1882 Prof. G. T. Brown published, in the *Journal of the Royal Agricultural Society of England*, an article in which he comes to the conclusion that, as a general thing, the views of the breeders cannot be relied upon, and that the recognition of the age from the teeth is still the best and surest. In June, 1886, the Executive Committee of the Fat-Stock Show at Berlin preferred similar complaints, and requested the Minister of Agriculture to introduce new experiments at the veterinary schools and agricultural experiment stations in Germany, to ascertain whether the signs of age from dentition, sexual development, and growth of horns can appear at an earlier time in our precocious breeds than hitherto believed. Accordingly, Prof. A. Nehring, of Berlin, published in the *Landwirtschaftliche Jahrbucher* of 1888, a series of new dentition tables for pigs, as a result of his studies and investigations upon a collection of one hundred and thirty-one skulls of different kinds of pigs, at the museum of the Royal Agricultural School at Berlin.

Having seen and examined parts of this collection, I will undertake to demonstrate, with the guide of the above-mentioned tables, together with my own experience and observation while practicing in breeding establishments, that our practical men have been quite right in many cases, and that the doubts to which reference has been made are not without foundation."

The pig, like the other animals, has two sets of teeth,—the temporary or milk teeth and the permanent ones.

"There exists a remarkable difference in the time occupied by the teeth in cutting their way through the gum and appearing on its surface, while the mode of succession remains unchanged. But it must be remembered that the dentition tables, still referred to in modern books for the practical pig breeder, are based upon observations made in times when the common pig was raised, or, perhaps, a breed more or less improved by English stock, and fed in the old fashioned style. Variations into early maturity were then described as abnormal; but as soon as the pure breeding of the favorites of our day commenced, Berkshire, Poland, China, *et al.*, and we applied to them scientific feeding, we forced the animals into entirely new and artificial conditions, revealing the hitherto unknown physiological laws of early maturity."

The periods of age which can be determined by the dentition are divided into:

1. Eruption of the temporary teeth.
2. Eruption of the permanent teeth.
3. Wearing of the permanent teeth, after 2½ or 3 years, an age attained but by few hogs.

These periods vary slightly, and when a latitude of time is given it is understood that the shorter is for precocious pigs and the longer for those races which approach the primitive pig.

### First Period—Eruption of the Temporary Teeth.

*At Birth.*—The pig is born with eight teeth,—the corner incisors of each jaw and the tusks. These teeth resemble each other very much, and probably serve to aid the tongue in sucking.

*Four to Fourteen Days.*—In the first two weeks the second upper molar and the third lower molar appear.

*Two to Five Weeks.*—During the next three weeks the second lower molar, the third upper molar, and the pinchers of both jaws accomplish their eruption.

*Five Weeks to Three Months.*—At about six weeks the intermediate incisors of the lower jaw appear, which are followed in two weeks by the intermediate incisors of the upper jaw.

Fig. 2.

Skull of a three-month old pig with full milk dentition.

*At three months* the eruption of the temporary teeth is completed, and during the next few months the teeth become more or less worn, according to the character of food upon which the animal is nourished.

### Second Period—Eruption of the Permanent Teeth.

*About Six Months.*—Before the first half-year the first permanent molar (fifth) and the first premolar (wolf-tooth) in each jaw appear.

*Nine Months.*—Within a month of three quarters of a year the corner incisors and the tusks make their eruption, followed shortly by the second molars (sixth) of both jaws.

*One Year.*—At one year the lower pinchers protrude from the gums, preceding the superior pinchers by two to three months. At about this time, thirteen to fourteen months, the third and fourth premolars appear, followed a month later by the second premolar.

*One and a Half Years.*—At eighteen months the intermediate incisors of both jaws make their appearance, and simultaneously the eruption of the third molar (seventh) takes place.

*After Two Years.*—The incisor teeth become used with great irregularity, according to the nature of the food; the tusks increase in length and give some approximation of their age, but insufficiently to be accurate.

---

### TABLE OF DENTITION OF THE HOG. (SCHWARTZKOPFF.)

#### MILK DENTITION.

| Teeth. | Precocious Pigs. | Normal Time of Appearance. | Primitive Pigs. | Simonds. |
|---|---|---|---|---|
| Corners and Tusks. | Present at birth. | Present at birth. | .............. | At birth. |
| Pinchers.............. | 2 weeks. | 3 to 4 weeks. | 5 weeks. | 1 month. |
| Intermediate ; | | | | |
| Upper jaw.......... | 8 weeks. | 12 weeks. | 16 weeks. | } 3 months |
| Lower jaw.......... | 5 weeks. | 8 weeks. | 12 weeks. | |
| 1st molar............. | 5 weeks. | 7 weeks. | 9 weeks. | |
| 2d molar: | | | | |
| Upper jaw.......... | 4 days. | 8 days. | 14 days. | |
| Lower jaw.......... | 2 weeks. | 3 to 4 weeks. | 5 weeks. | |
| 3d molar: | | | | |
| Upper jaw.......... | 2 weeks. | 3 to 4 weeks. | 5 weeks. | |
| Lower jaw.......... | 4 days. | 8 days. | 14 days. | |

PERMANENT DENTITION.

| | | | | |
|---|---|---|---|---|
| Pinchers | 11 months. | 12 months. | 14 months. | 12 months. |
| Intermediate | 16 months. | 18 months. | 21 months. | 18 months. |
| Corners | 7½ to 8 months. | 9 months. | 10 months. | 9 months. |
| Tusks | 3½ months. | 9 months. | 10 months. | 9 months. |
| 1st premolar | 2 to 3 months. | 5 months. | 6 months. | |
| 2d premolar | 13 months. | 14 to 15 months. | 16 months. | |
| 3d premolar | 12 months. | 13 to 14 months. | 15 months. | |
| 4th premolar | 12 months. | 13 to 14 months. | 15 months. | |
| 1st molar | 2 months. | 5 months. | 6 months. | |
| 2d molar | 7 to 8 months. | 9 to 10 months. | 12 to 14 mos. | |
| 3d molar | 17 months. | 18 to 19 months. | 20 to 22 mos. | |

The following explanations of the causes influencing the variations in the dentition of the pig is given by Schwartzkopff :—

"The question now arises as to what may be regarded as the cause of this early dentition in modern pigs. We know that our present method of feeding causes a rapid development of the whole body, including, of course, the head. As the teeth could not possibly grow without a corresponding growth of the jaws, that produce them, we must conclude that the development of the skull is the primary cause or driving force in their development. Unconsciously, the modern feeder has produced here some highly interesting facts instructive to natural science at large. Hitherto zoologists have been of the opinion that the form of skull of a fixed species is unchangeable from generation to generation, we may say for thousands of years. This is correct as long as we think of individuals raised in the freedom of nature and under natural and similar circumstances. But domestication, with its forced feeding and breeding for various demands, has brought about unexpected changes in many respects, and is now evident that the form of skull does not rest merely upon heredity. Only a predisposition to a certain form of skull is transferable from parents to their offsprings, but whether exactly the same form will be transmitted depends to a greater extent upon the nutrition, and but little less upon the employment of the muscles of the head and neck. It is not only important that the nourishment be abundant and well selected, but it is also necessary that the individual be in a healthy condition, and his digestive apparatus in such working order as to be able to utilize the offered food equally well. This is plainly seen by comparing skulls from animals which were healthy and growing vigorously with those which received the same advantages of nutrition, but were suffering with a chronic disease. Continued weakness, caused either by disease or insufficient food, produces a long, slender skull, while the skull from a strong pig shows a remarkable expansion in its latitude and altitude. The following reproductions, taken from originals in the agricultural museum at Berlin, will illustrate this point :—

FIG. 3.
Skull of a three-month-old pig which died from tuberculosis.
(Half natural size.)

FIG. 4.
Skull of two-month-old healthy and well-fed pig.
(Half natural size.)

"Besides the nutrition influence, a strong or weak muscular action plays an important part in the production of form. The pulling and pressure of muscles extensively used for certain purposes, especially those of the head and neck, will give the head a characteristic shape. Pigs which are prevented from rooting will acquire a short, high, and rounded head, while those which are forced to root to secure a portion of their food will develop a long and slender form of head. If we force both experiments to the greatest degree possible, we shall produce those extremes which distinguish the wild pig from our improved races. That this is true is proven by the fact that when our domestic hogs are returned to absolute liberty, it will require but a few generations to reproduce the original skull of the wild pig. And, *vice versa*, we have called into existence, from the primitive hog, all those different representative types of our day by careful and continued selection, gradual assortment, and particular attention to the desired qualities of form, size, etc.

The striking difference between the skull of a primitive hog and a modern one is seen in the following illustration :—

FIG. 5.

Skull of a full-grown primitive Texas boar.

FIG. 6.
Skull of a full-grown sow of the small Yorkshire breed.

"The pig has, perhaps, the most elastic and changeable organization of any of our domestic animals. It also has the advantage of being able to digest all kinds of food as an omnivorous animal, and last, though not least, it multiplies more rapidly than any domestic animal,—even the sheep. Therefore, it has been at all times regarded, and promptly too, as the animal *par excellence* for experiments in breeding, and the pig is the best example of what men have accomplished in the production of animals.

"Drawing, now, the conclusions from the above examinations, I shall summarize them in the following theses :—

"1. The order of succession of the teeth in our precocious pigs remains the same as in the primitive hog.

"2. The times when the teeth appear are variable, according to the race, feeding, and health. The same breeds, raised under the same conditions, will show the same appearance.

"3. The form of the skull depends upon nutrition, health, and more or less employment of certain muscles of the head and neck."

---

## Figures and Tables

**Fig. 1. f1:**
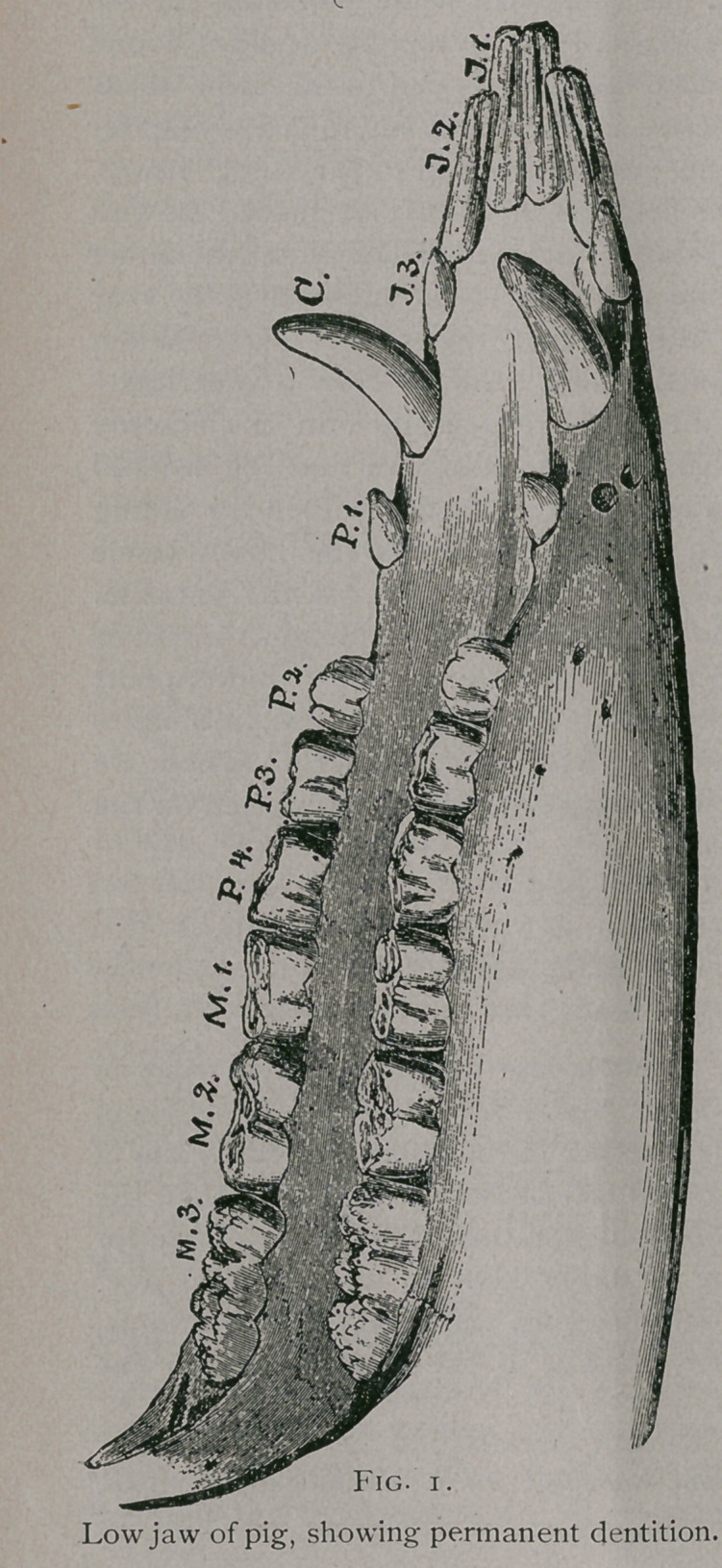


**Fig. 2. f2:**
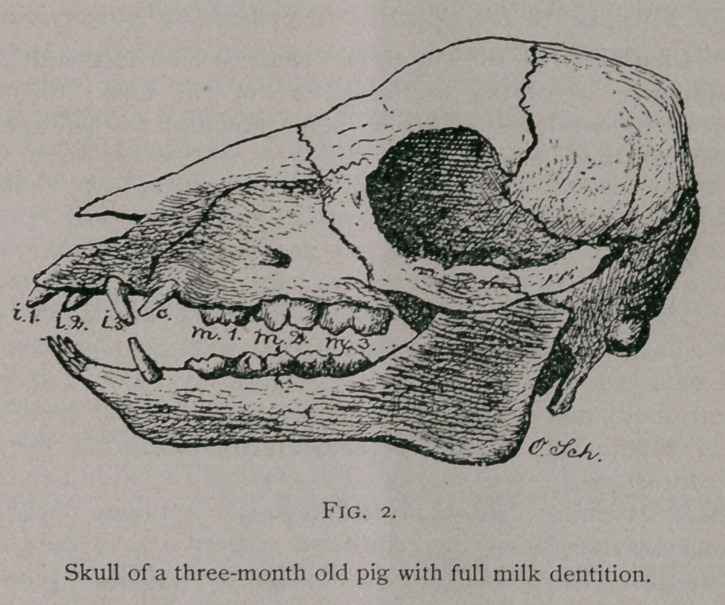


**Fig. 3. f3:**
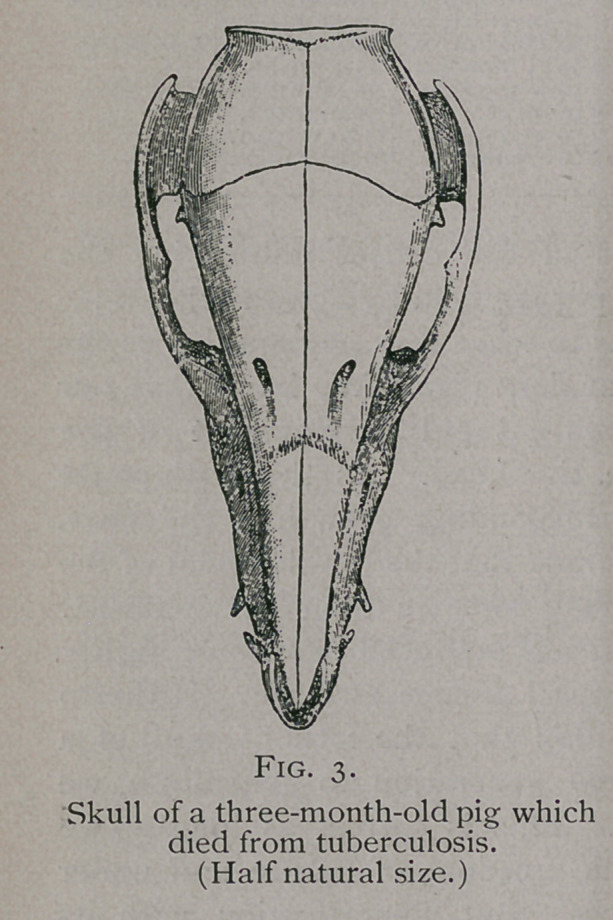


**Fig. 4. f4:**
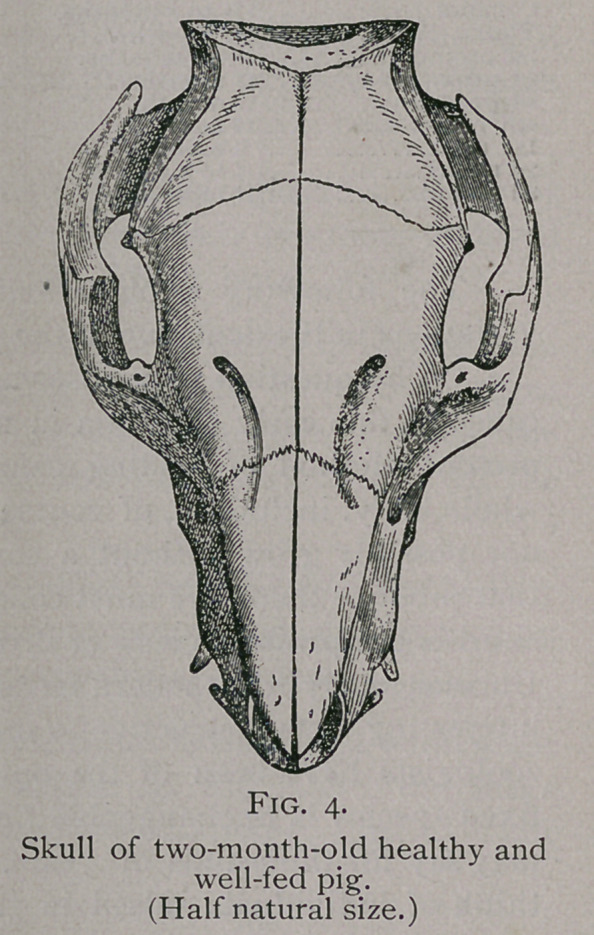


**Fig. 5. f5:**
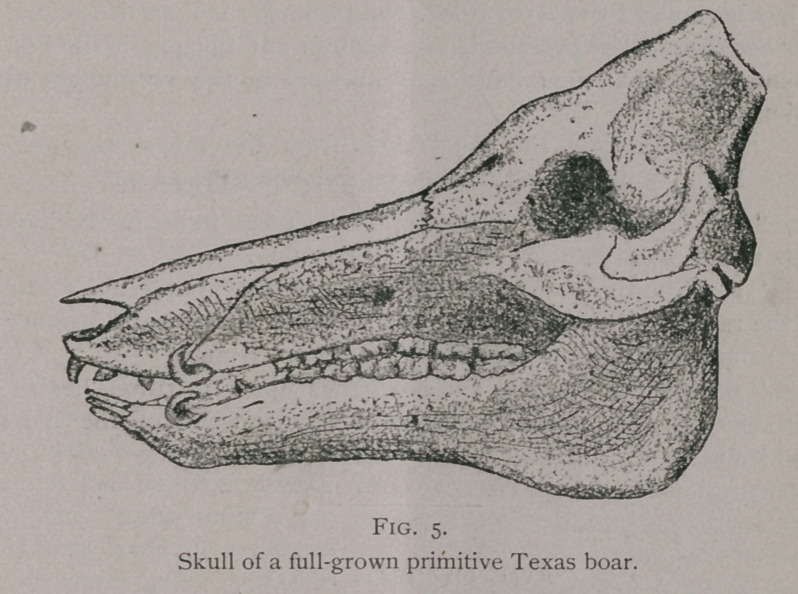


**Fig. 6. f6:**